# Performance of Class I composite restorations when polished immediately or after one-day water storage

**DOI:** 10.1371/journal.pone.0183381

**Published:** 2017-08-17

**Authors:** Masao Irie, Yukinori Maruo, Goro Nishigawa

**Affiliations:** 1 Department of Biomaterials, Okayama University Graduate School of Medicine, Dentistry and Pharmaceutical Science, Okayama, Japan; 2 Occlusion and Removable Prosthodontics, Okayama University Hospital, Okayama, Japan; Queen's University at Kingston, CANADA

## Abstract

This study investigated the effects on gap formation in Class I restorations (observed by vertical and horizontal forms of inspection) and on the mechanical properties of nine resin composite filling materials when the restorations were subject to finishing immediately after setting or after one-day water storage. Class I restorations with resin composite fillings were polished either immediately (3 min) after setting or after one-day water storage. Interfacial gap formation (observed by vertical inspection) was assessed using 14 gap measurement points along the interface between the restoration and cavity walls and floor (*n* = 10 per resin composite; total points measured per time point = 140). For marginal gaps formed at cavosurface margins in Class I cavities and in Teflon molds, marginal gap formation (observed by horizontal inspection) was assessed by measuring the maximum gap-width and opposing width (if any). Effects on mechanical properties were assessed by measuring shear bond strengths to enamel and dentin, flexural strength and modulus. After one-day storage, marginal gap-widths in Class I restorations were significantly decreased for all composites, alongside a significant increase in shear bond strengths to enamel and dentin, flexural strength and modulus. Resin composite-filled Class I restorations which were polished after one-day delay presented lower gap formation compared with finishing immediately after setting.

## Introduction

Polymerization shrinkage occurs during the early stage of polymerization of light-activated dental composites. This phenomenon adversely affects interfacial adaptation and bonding to tooth structure because the shrinkage forces generated can disrupt the bond to cavity walls and result in gap formation. As for the adhesive systems used to bond the restorative filling materials to tooth structure, their issues of flow ability, polymerization shrinkage and the resulting destructive shrinkage stress further contribute to gap formation in resin composite restorations [1–8]. Filling technique and composite type may also affect the adhesion of composites [9, 10]. Compromised marginal integrity at the resin-tooth interface will lead to bacterial penetration, and subsequently pulpal damage and postoperative sensitivities [1, 5, 6]. One way to predict the clinical success of dental composite restorations *in vitro* is to evaluate marginal adaptation [11–13]. In butt-joint cavities restored with an adhesive system and a resin composite filling, the magnitude of interfacial gaps formed is dictated by these factors: (1) adhesive forces between the restorative material and cavity walls; (2) degrees of volumetric contraction of filling and luting materials; and (3) flow properties of filling and luting materials [4, 11–13].

Self-etch primer adhesive systems and all-in-one adhesives vary in their acidity because of differences in the composition and concentration of polymerizable acids and/or acidic resin monomers [9–15]. Compared with adhesive systems that need separate acid-conditioning and rinsing steps, these adhesives require less complicated, less time-consuming, and less technique-sensitive application procedures. Clinically, interfacial bonding provided by these contemporary adhesive systems must be sufficiently strong to withstand masticatory and parafunctional stresses in the wet, warm oral environment. Therefore, the mechanical properties of both resin composite restorative filling materials and luting agents directly influence the marginal seal or gap formation, which then affects the clinical success of dental composite restorations [14, 16, 17].

The mechanical properties of restorative filling materials and luting agents have been evaluated using *in vitro* flexural testing [14, 17]. In our previous studies [14, 17], dental restorations which were polished after one-day storage following light activation had improved shear bond strength and flexural properties, and thus improved marginal integrity. Similarly for luting agents, their shear bond strengths to dentin and flexural moduli increased after one-day storage, coupled with markedly decreased incidence of interfacial gaps [17].

In the present study, Class I cavities were restored with nine different commercial resin composite filling materials and self-etch adhesives. At 3 min after the start of light activation and after one-day storage, shear bond strengths to enamel and dentin and flexural properties of resin composite filling materials were measured. Gap formation after immediate and one-day-delayed polishing was assessed to investigate the effects of these mechanical properties on interfacial gap formation. The hypothesis to be tested in this study was that premature finishing would significantly reduce gap formation as opposed to delayed finishing.

## Materials and methods

### Human teeth

Human premolars, extracted for orthodontic reasons, were used in this study. After extraction and cleaning, the teeth were immediately stored in cold distilled water at 4°C for 1–2 months before use.

The research protocol of this study was approved by the Ethics Committee of Okayama University Graduate School of Medicine, Dentistry and Pharmaceuticals Sciences, and Okayama University Hospital (No. 1508–007).

### Light-activated resin composite filling materials and self-etch adhesives

Nine light-activated resin composite filling materials for premolar restorations were selected for this study. Details of these resin composite filling materials are listed in Table 1, and those of their accompanying adhesive systems are listed in Table 2. These resin composite restorative materials and adhesives systems were selected because they were the major restorative products used by dentists and thus provided a comprehensive, clinically relevant range of values for the parameters to be investigated in this study.

**Table 1:** Light-activated restorative materials investigated in this study.

Product	Composition	Manufacturer	Lot No.
QuiXX	Silica nanofiller (86 wt%, 66 vol%), Bis-EMA, UDMA, TEGDMA, TMPTMA	Dentsply/Caulk, Milford, DE, USA	503000635
Filtek P60	Zirconia/silica (83 wt%, 61 vol%) Bis-GMA, UDMA, Bis-EMA, Photo initiators, stabilizers	3M ESPE, St. Paul, MN, USA	3TC
Herculite XRV	Barium silica glass (79 wt%, 59 vol%), Bis-GMA, TEGDMA, EBPADMA	Kerr, Orange, CA, USA	112330
Tetric N-Ceram	Bariumglass filler, Ytterbiumtrifluoride, Mixed oxide(63.5 wt%, 55–57 vol%), Prepolymer (17%) UDMA, Bis-EMA, Bis-GMA, Photo initiators	Ivoclar Vivadent AG, Schaan, Liechtenstein	KO4764
Gradia Direct P	Silica powder, Prepolymerized filler, Fluoro-aluminosilicate-glass (79 wt%, 65 vol%), UDMA, Dimethacrylate, Pigment, Photo initiators	GC, Tokyo, Japan	403301
BEAUTIFIL II	S-PRG filler, multi-functional glass filler, Ultra-fine filler (83.3 wt%, 68.6 vol%), Bis-GMA, TEGDMA, UDA, Photo initiators	Shofu, Kyoto, Japan	110615
EPIC-AP	Barium glass filler, TMPT reactive filler (82 wt%, 64 vol%), Dimethacrylates, Photoinitiator, Stabilizer	Sun Medical, Moriyama, Japan	MX2F
Estelite Sigma	Silica/zirconia filler (82 wt%, 71 vol%), Bis-GMA, TEGDMA, Bis-MPEPP, Photo initiators	Tokuyama Dental, Tokyo, Japan	011K2
Clearfil AP-X	Silanater glass ceramics, Surface treated alumina micro filler (85.5 wt%, 71.0 vol%), Bis-DGMA, TEGDMA, Hydrophobic dl-Camphorquinonearomatic dimetnacrulate,	Kuraray Medical, Kurashiki, Japan	1121AA

Bis-EMA: Bisphenol A ethoxyl methacrylate, Bis-DGMA: Bisphenol A diglycidyl mentacrylate, DMA: Urethane dimethacrylate, TEGDMA: Tri-ethylene-glycol dimethacrylate, TMPTMA: Trimethylolpropane trimetharylate, Bis-GMA: Bisphenol A glycidyl methacrylate, TMPT: Trimethylolpropane trimetharylate, EBPADMA: Ethoxylated bis-phenol-A-dimethacrylate, UDA: Urethane diacrylate, S-PRG: Surface reaction type pre-reacted glass-ionomer filler, Bis-MPEPP: 2,2-Bis(4-methacryloyloxypolyethoxyphenyl)propane

**Table 2:** Self-etch adhesives and their components.

Adhesive	Composition and surface treatment	Manufacturer	Batch No.
Xeno IV	Polymerizable organophosphate monomer, Polymerizable organocarboxlic acid monomer, Polymerizable tri/dimethacrylate resin, Light cure initiator, Stabilizer, acetone, Xeno IV (20 s)–air–light (10 s)	Dentsply/Caulk, Milford, DE, USA	41229
Adper Prompt L-Pop	Methacrylated phosphoric acid ester, Water, Phosphine oxide, Stabilizer, Fluoride complex, Adper Prompt L-Pop (15 s)–air–light (10 s)	3M ESPE AG, Seefeld, Germany	177396
OptiBond All-in-One	GPDM, Co-monoers, Water, Acetone, Ethanol, Phototoinitiator, Nano-filler, Fluorid-releasing fillers, OptiBond All-in-One (20 s)–air–OptiBond All-in-One (20 s)–Strong air–light (10 s)	Kerr, Orange, CA, USA	436167
AdheSE One VivaPen	Bis-acrylamide derivative, Water, Bis-methacrylamide dihydrogenphosphate, Bis-methacrylamide dihydrogenphosphate, AdheSE One VivaPen (10 s)–air–light (10 s)	Ivoclar Vivadent AG, Schaan, Liechtenstein	R 320611
G-Bond	UDMA, 4-MET, Silica filler, Phosphoric acid ester monomer, Acetone, Water, Phototoinitiator, G-Bond (10 s)–strong air–light (10 s)	GC, Tokyo, Japan	404021
FL-BOND II	Primer: Carboxylic acid monomer, Phosphonic acid monomer, water, solvent, Initiator, Bond: S-PRG filler, UDMA, TEGDMA, HEMA, Photo-initiator, Primer (10 s)–air–Bond–light (10 s)	Shofu, Kyoto, Japan	787T2060712
Brush & Bond	Liquid: 4-META, UDMA, Monomethacrylates, water-acetone, Photo initiator, Stabilizer, Cata-sponge: Sopdium p-toluenesulfinate, Aromatic amine, AQ Bond Plus (20s)–gentle air (15s)–strong air (5s)–light (10 s)	Sun Medical, Moriyama, Japan	FW1
Tokuyama Bond Force	A: Phosphoric acid Monomer, MAC-10, Bis-MPEPP, MMA, B: HEMA, MMA, Water, Fluoroaluminosilicateglass, Borate catalyst, Tokuyama Bond Force (A+B, 20s)–air–light (10 s)	Tokuyama Dental, Tokyo, Japan	1
Clearfil tri-S Bond	MDP, HEMA, Bis-DGMA, Water, Ethanol, dl-Camphorquinone, Silanated colloidal silica, Tri-S Bond (20s)–air–light (10 s)	Kuraray Medical, Kurashiki, Japan	11194

HFGA-GDM: Hexafluoroglutaric anhydride-Glycerodimethacrylate adduct, GPDM: Glycerophosphatedimethacrylate, UDMA: Urethane dimethacrylate, 4-MET: 4—methacryloxyethyl trimellitic acid, HEMA: 2-Hydroxyethyl methacrylate, TEGDMA: Tri-ethylene-glycol dimethacrylate, 4-META: 4—methacryloxyethyl trimellitate anhydrine, S-PRG: Surface reaction type pre-reacted glass-ionomer filler, MAC-10: 11-methacryloyloxy-1, 1-undecanedicarboxylic acid, Bis-MPEPP: 2,2-Bis(4-methacryloyloxypolyethoxyphenyl)propane, MMA: methylmethacrylate, MDP: 10-Methacryloyloxydecyl dihydrogen phosphate, Bis-GMA: Bisphenol A glycidyl methacrylate, Bis-DGMA: Bisphenol A diglycidyl mentacrylate

Tooth preparation procedures, mixing, and handling were carried out according to manufacturers’ recommendations (Table 2). A visible light curing unit (New Light VL-II, GC, Tokyo, Japan; fiber optic tip diameter: 8 mm) was used to irradiate the light-activated materials for 20 sec. Using a radiometer (Demetron/Kerr, Danbury, CT, USA), light intensity was checked immediately before the application of each adhesive resin and composite filling material. During light curing, light intensity was maintained at 450 mW/cm^2^.

### Preparation and restoration of Class I cavities

**Fig 1:**
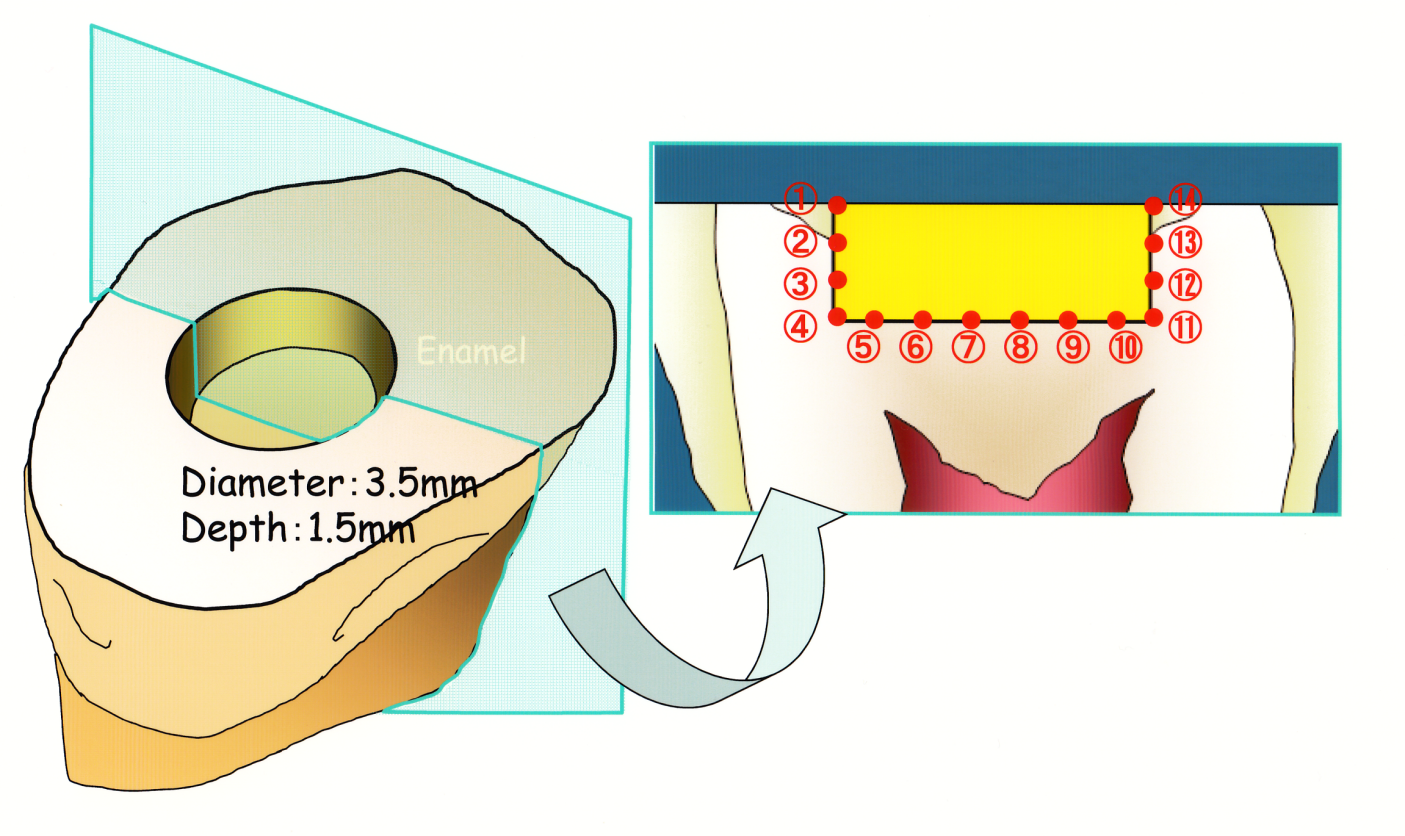
Schematic illustration of 14 measurement points and standardized cavity dimensions.

Each human premolar tooth was embedded in a slow-setting epoxy resin (Epofix, Struers, Copenhagen, Denmark). A Class I cavity was prepared in the enamel surface using a tungsten carbide bur (200,000 rpm) and a fissure bur (8,000 rpm) under wet conditions. Cavity dimensions were standardized as follows: 3.5 mm diameter and 1.5 mm depth (Fig 1, C-factor = 2.72) [18]. Cavosurface walls were finished to a butt joint. Cavity design used in the present study differed from clinical Class I cavities in that cavity corners were at right angles for the purpose of obtaining constant-volume models. One cavity was prepared in each of 180 teeth (9 resin composite restorative materials × 2 polishing times × 10 replicates). A single operator (Masao IRIE) carried out all the procedures (cavity preparation, adhesive system application, restoration and polishing).

Cavity walls and surrounding enamel margin were pre-treated according to manufacturers’ instruction (Table 2). Each cavity was filled in with only one increment of each restorative material using a syringe tip (Centrix C-R Syringe System, Centrix, Connecticut, USA) and then covered with a plastic strip. Composite filling was cured by light irradiation.

### Inspection for interfacial gaps between restoration and cavity walls (Vertical inspection)

Immediately after setting (*i*.*e*., at 3 min after the start of light activation) or after storage in distilled water at 37°C for one day, excess composite restorative material on each restoration surface was removed by wet grinding on #600 carborundum paper. This was followed by polishing with linen and an aqueous alumina slurry (Alpha Micropolish, Buehler, Chicago, IL, USA). Then, distilled water was used for rinsing to avoid desiccation and erosion.

Each tooth was sectioned in a buccolingual direction through the center of the restoration with a low-speed diamond saw (IsoMet, Buehler, Lake Bluff, IL, USA). The presence or absence of interfacial gaps [14] was inspected and measured at 14 points, labelled as Point #1 to Point #14, each 0.5 mm apart (Fig 1), along the interface between the restoration and cavity walls (*n* = 10; total points measured per resin composite = 140). Inspection and measurements were carried out using a measurement microscope at ×1,000 magnification (Measurescope MM-11, Nikon, Tokyo, Japan), with the cavity walls and cavity floor of each half of the sectioned tooth facing up. At 3 min after the start of light activation or after one-day storage, the number of gaps at each measurement point (Point #1 to Point #14) in each tooth was summed up for each resin composite restorative material [14, 15].

### Inspection for marginal gap-widths between restoration and cavosurface margin (Horizontal inspection)

One cavity was prepared in each of 180 teeth (9 resin composite restorative materials × 2 polishing times × 10 replicates), using the same, standardized cavity dimensions and preparation and restoration procedures described in **subsection 2.3** for Class I cavities. Immediately after light-curing, excess composite restorative material on each restoration surface was removed by wet grinding on #600 carborundum paper. This was followed by polishing with linen and an aqueous alumina slurry (Alpha Micropolish, Buehler, Chicago, IL, USA).

**Fig 2:**
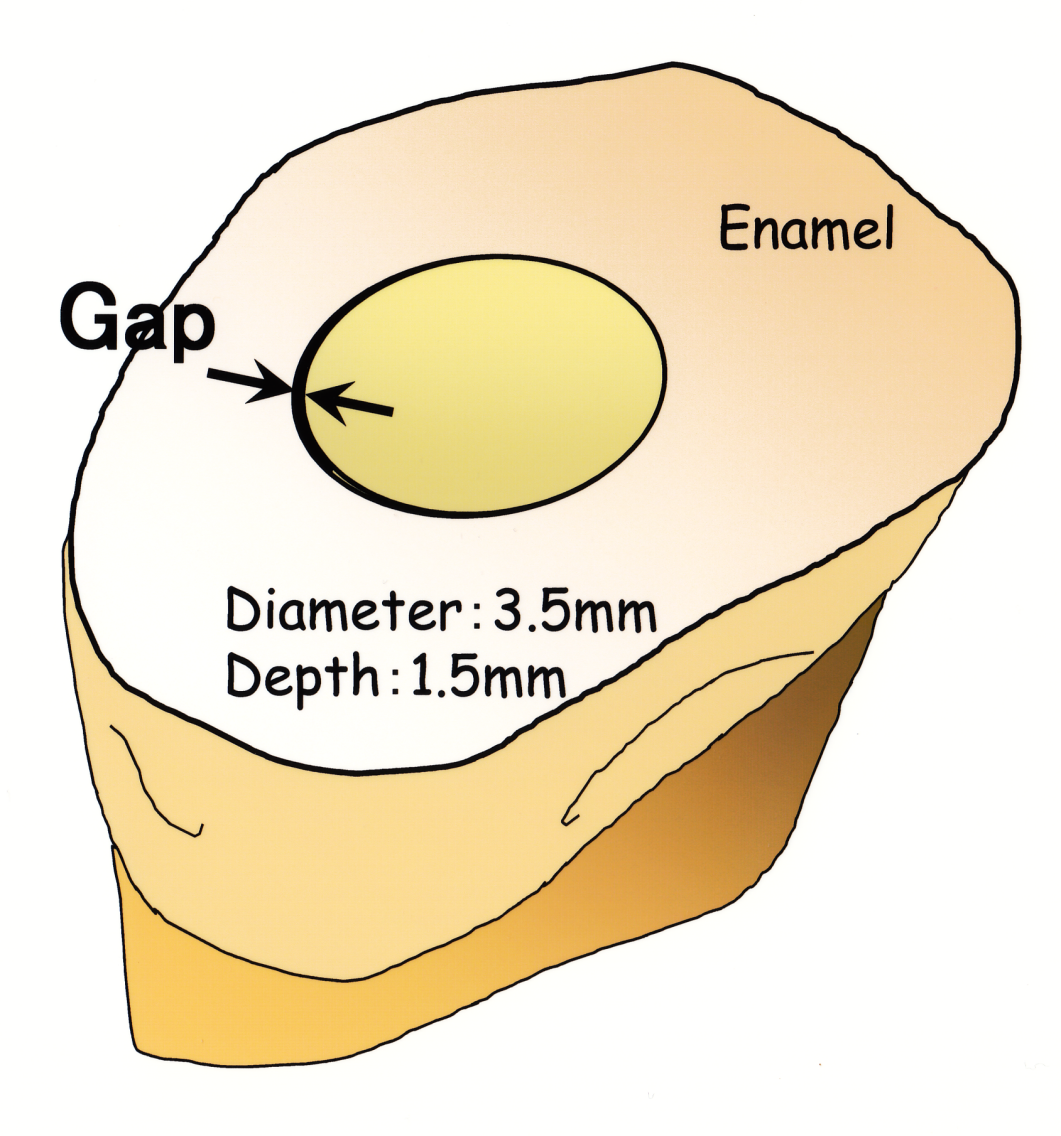
Schematic illustration of marginal gap-widths.

Cavosurface margin on the enamel surface of each restoration (Fig 2) was inspected using a measurement microscope at ×1,000 magnification (Measurescope MM-11, Nikon, Tokyo, Japan). Maximum gap-width and opposing width (if any) between the restorative material and enamel margin was recorded [11, 12]. Maximum gap-widths in each tooth (*n* = 10) were summed up for each resin composite restorative material.

### Measurement of marginal gaps in Teflon cavity (Horizontal inspection)

Teflon molds were chosen for this study because they do not react with resin composites, allowing them to freely shrink in the cavities during polymerization.

Prepared Teflon molds (*n* = 10), each of 1.5 mm depth and 3.5 mm diameter, were placed on silicone oil-coated glass plates and filled with a resin composite restorative material using a syringe tip. Each mold was covered with a plastic strip until set. Polymerization shrinkage was measured immediately after setting, and hygroscopic expansion (if any) was measured after one-day storage in water. Sum of the maximum gap-width and opposing gap width (if any) was expressed as the marginal gap of the resin composite material in the Teflon cavity [12].

### Measurement of shear bond strengths to enamel and dentin

Buccal surfaces were wet-ground with silicon carbide abrasive papers up to 1000 grit until a flat enamel or superficial dentin area of at least 4 mm diameter was exposed (*n* = 10 per resin composite). The flat exposed surface was treated according to manufacturers’ instructions (Table 2).

A split Teflon mold with a cylindrical hole (3.6 mm diameter, 2 mm height) was clamped to the prepared enamel or dentin surface in a mounting jig. With the cylindrical hole centrally positioned over the 4-mm-diameter prepared tooth surface, the Teflon mold was filled with a restorative material using a syringe tip (Centrix C-R Syringe System, Centrix, CT, USA). It was covered with a plastic strip, and the composite filling material was cured by light irradiation.

At 3 min after the start of light irradiation or after one-day storage, a shear force was applied using a universal testing machine (Autograph DCS-2000, Shimadzu, Kyoto, Japan) at a crosshead speed of 0.5 mm/min. The force was transmitted *via* a flat (blunt) 1-mm-thick shearing blade at a perpendicular direction to the load. Stress at failure was calculated and recorded as the shear bond strength. Failed specimens were examined under a light microscope at ×4 magnification (SMZ-10, Nikon, Tokyo, Japan) to ascertain the fracture mode (three categories: adhesive failure, mixed failure, or cohesive failure in resin), and determine the total number of adhesive failures [12, 17].

### Measurement of flexural strength and flexural modulus of elasticity

Resin composite specimens for flexural testing (*n* = 10 per resin composite) were filled into Teflon molds (25×2×2 mm^3^) and light-cured in three overlapping sections, each cured for 20 sec.

Immediately after setting (*i*.*e*., at 3 min after start of light irradiation) or after one-day storage, resin composite specimens were subject to three-point bending test with a 20-mm span and a load speed of 0.5 mm/min (Model 5565, Instron, Canton, MA, USA), as outlined in ISO 9917–2 (1996). Flexural strength was determined from the highest stress experienced at fracture, and flexural modulus was accordingly calculated using an accompanying software (Series IX software, Instron, Canton, MA, USA).

### Statistical analysis

Data were statistically analyzed using Mann-Whitney U test, Tukey’s test [12–14, 17, 19], and *t*-test. Significant difference was set at *p*<0.05.

All procedures, except for testing, were performed in an air-conditioned room at 23±0.5°C and 50±2% R.H.

## Results and discussion

### Interfacial gaps between restoration and cavity walls

Tables 3 and 4 (using the measurement points #1 to #14 labeled in Fig 1) present the data for the summed interfacial gaps observed in Class I restorations at two time points: immediate *versus* after one-day storage. Data mean was not used because many specimens had no gaps. Therefore, the overall sum of data was used instead.

**Table 3:** Effect of polishing time on interfacial gap formation between Class I restorations and cavity walls and floor.

Product	Number of specimens showing gaps			
	Medial	Bottom	Distal	Alpha value*
Polishing time	1 2 3 4	5 6 7 8 9 10	11 12 13 14	
Composite + pretreating agent				
QuiXX + Xeno IV				
Immediately	6 2 0 3	1 0 0 1 1 0	3 0 3 7	27
After one-day storage	3 2 0 1	0 0 1 0 0 1	3 0 0 3	14
				<0.05
P-60 + Adper Prompt L-Pop				
Immediately	6 2 3 2	2 0 1 0 0 3	2 1 2 8	32
After one-day storage	5 0 1 2	1 0 0 2 1 2	1 0 1 6	22
				<0.05
Herculite XRV + AIO Adhesive				
Immediately	9 2 0 2	1 1 1 4 0 1	4 0 1 9	35
After one-day storage	6 0 2 2	0 1 1 0 0 1	1 0 0 6	20
				<0.05
Tetric EvoCeram + AdheSE				
Immediately	6 2 0 4	1 0 0 1 0 1	3 2 2 9	31
After one-day storage	6 0 0 3	1 0 0 0 2 0	1 0 0 7	20
				<0.05
Gradia Direct P + G Bond				
Immediately	5 2 1 4	1 0 1 0 2 2	2 2 2 8	32
After one-day storage	3 0 0 3	0 0 1 1 0 0	1 0 2 5	16
				<0.05
BEAUTIFIL II + FL-BOND II				
Immediately	6 3 0 4	0 1 1 1 0 0	6 1 2 6	31
After one-day storage	5 1 0 2	2 0 0 0 1 0	2 0 0 5	18
				<0.05
EPIC-AP + Brush & Bond				
Immediately	8 2 1 3	2 0 2 2 0 2	4 2 3 7	38
After one-day storage	5 0 0 2	0 1 0 0 1 0	1 0 0 5	15
				<0.05
Estelite Sigma + One Up Bond F Plus				
Immediately	7 2 1 4	0 2 0 2 0 1	5 0 2 7	33
After one-day storage	4 0 1 2	0 0 2 0 0 2	2 0 0 4	17
				<0.05
Clearfil AP-X + Clearfil tri-S Bond				
Immediately	6 2 1 1	0 0 2 0 1 0	2 2 4 8	29
After one-day storage	2 1 0 2	0 1 2 0 0 0	2 2 0 3	15
				<0.05

n = 10 (total measurement points, 1–14 = 140)

*: Significantly different according to Mann-Whitney U test between the two conditions (p = 0.05).

For all resin composite products, significant differences (*p*<0.05) were observed in the number of gaps formed between the immediate time point and after one-day storage At each time point and for all resin composites, points #1 and #14 presented the highest number of gaps. Points #4 and #11 at the cervical area also showed a few gaps. Points #5 to #9 at the cavity floor showed almost no gaps at both time points.

**Table 4:** Effect of polishing time on interfacial gap in Class I restorations corresponding to Table 3.

Restorative material	Sum of interfacial gaps for 10 specimens (n)			Alpha value *
	Immediately	After one-day storage	Change (%) ^#^	
QuiXX + Xeno IV	27 A	14 B	-48	<0.05
P-60 + Adper Prompt L-Pop	32 A	23 B	-31	<0.05
Herculite XRV + AIO Adhesive	35 A	20 B	-43	<0.05
Tetric EvoCeram+ AdheSE	31 A	20 B	-35	<0.05
Gradia Direct P + G Bond	32 A	16 B	-50	<0.05
BEATIFIL II + FL-BOND II	31A	18 B	-42	<0.05
EPIC-AP + Brush & Bond	38 A	15 B	-61	<0.05
Estelite Sigma + One Up Bond F Plus	33 A	17 B	-48	<0.05
Clearfil AP-X + Clearfil tri-S Bond	29 A	15 B	-48	<0.05

n = 10 (total measurement points, 1–14 = 140), Means with the same letters (A, B) were not significantly according to Tukey‘s test (p>0.05, non-parametric [16–18]).

*: Significantly different according to Mann-Whitney U test between the two conditions (p = 0.05).

^#^: Percentage to the immediate condition

For all resin composite products, significant differences (*p*<0.05) were observed in the number of gaps formed between the immediate time point and after one-day storage (ranging from –31% to –61%). Immediately after setting, total number of interfacial gaps formed in each composite ranged between 27 and 38, with no statistically significant differences among the composites. After one-day storage, total number of gaps found in each composite ranged between 14 and 22, with no statistically significant differences among the composites.

### Marginal gap-widths between restoration and cavosurface margin

Table 5 presents the data for the summed marginal gap-widths observed at cavosurface margin on the enamel surface at two time points: immediate *versus* after one-day storage.

**Table 5:** Effect of polishing time on marginal gap-width at cavosurface margin on enamel surface.

Restorative material	Sum of marginal gap-width for 10 specimens (μm)			Alpha value *
	Immediately	After one-day storage	Change (%) ^#^	
QuiXX + Xeno IV	64 (0)^a^ (2–9) ^b^ AB	5 (8)^a^ (0–3) ^b^ C	-92	<0.05
P-60 + Adper Prompt L-Pop	61 (0)^a^ (2–12) ^b^ AB	18 (5)^a^ (0–5) ^b^ C	-70	<0.05
Herculite XRV + AIO Adhesive	88 (0)^a^ (5–12) ^b^ A	24 (5)^a^ (0–7) ^b^ C	-73	<0.05
Tetric EvoCeram+ AdheSE	54 (0)^a^ (2–8) ^b^ AB	24 (5)^a^ (0–6) ^b^ C	-56	<0.05
Gradia Direct P + G Bond	46 (2)^a^ (0–8) ^b^ B	9 (7)^a^ (0–4) ^b^ C	-80	<0.05
BEATIFIL II + FL-BOND II	34 (3)^a^ (0–7) ^b^ B	8 (7)^a^ (0–4) ^b^ C	-76	<0.05
EPIC-AP + Brush & Bond	52 (0)^a^ (3–8) ^b^ AB	25 (5)^a^ (0–7) ^b^ C	-52	<0.05
Estelite Sigma + One Up Bond F Plus	45 (3)^a^ (0–9) ^b^ AB	19 (6)^a^ (0–7) ^b^ C	-58	<0.05
Clearfil AP-X + Clearfil tri-S Bond	48 (2)^a^ (0–8) ^b^ AB	29 (5)^a^ (0–8) ^b^ C	-40	<0.05

Means with the same letters (A-C) were not significantly different according to Tukey’s test (p>0.05, non-parametric [16–18]).

^a^: Number of specimens with no interfacial gaps.

^b^: Range of marginal gap-widths.

*: Significantly different according to Mann-Whitney U test between the two conditions (p = 0.05).

^#^: Percentage change when compared with the immediate condition

For all resin composites products, significant differences (*p*<0.05) were observed in the marginal gap-widths formed between the immediate time point and after one-day storage (ranging from –40% to –92%). Immediately after setting, summed marginal gap-widths (sum of maximum gap width and opposing width (if any)) of all composites ranged between 34 and 88 μm, and a few specimens of each resin composite product had no gaps. After one-day storage, summed marginal gap-widths ranged between 5 and 29 μm, with a few specimens of each resin composite product presenting no marginal gaps. There were no statistically significant differences among the resin composites after one-day storage.

### Marginal gap-widths in Teflon cavities

Table 6 presents the marginal gap-widths formed in Teflon cavities at two time points: immediate *versus* after one-day storage.

**Table 6:** Effect of polishing time on marginal gap-width in a Teflon mold (μm; mean (standard deviation)).

Restoration	Immediately	After one-day storage	Change (%) ^#^	p value ^a^
QuiXX	11.3 (2.8) A	11.1 (1.3) C	-2	NS
Filtek P60	11.5 (1.5) A	11.2 (2.0) C	-3	NS
Herculite XRV	16.1 (1.7) B	16.6 (1.6) D	3	NS
Tetric EvoCeram	10.6 (1.9) A	11.1 (2.1) C	5	NS
Gradia Direct P	11.0 (1.6) A	10.4 (2.1) C	-5	NS
BEAUTIFIL II	11.8 (1.4) A	11.7 (1.3) C	-1	NS
EPIC-AP	10.7 (0.8) A	10.6 (1.3) C	-1	NS
Estelite Sigma	10.9 (1.3) A	10.5 (1.6) C	-4	NS
Clearfil AP-X	11.8 (1.7) A	11.1 (2.0) C	-6	NS

n = 10

^a^: t-test

NS: No significant difference between two conditions (p>0.05). Means with the same letters (A-D) were not significantly different according to Tukey‘s test (p>0.05).

#: Percentage change when compared with the immediate condition

For all resin composite products, no significant differences (*p*>0.05) in marginal gap-width were observed between the immediate time point and after one-day storage (ranging from –6% to +5%). At each time point, no significant differences (*p*>0.05) were observed among all the resin composite products, except Herculite XRV.

### Shear bond strength to enamel

Table 7 presents the shear bond strengths to enamel. Significant differences (*p*<0.05) in shear bond strength were observed between the immediate time point and after one-day storage for all resin composite products (except Clearfil AP-X), ranging from +16% to +109%.

**Table 7:** Shear bond strengths to enamel (MPa; mean (standard deviation), number of adhesive failures).

Restoration	Immediately	After one-day storage	Change (%) ^#^	p value ^a^
QuiXX + Xeno IV	12.0 (2.5, 0) CDE	20.7 (3.5, 0) F	73	<0.05
P-60 + Adper Prompt L-Pop	14.2 (2.4, 0) BC	18.0 (4.4, 0) F	27	<0.05
Herculite XRV + AIO Adhesive	8.3 (2.6, 6) E	16.3 (4.4, 4) FG	96	<0.05
Tetric EvoCeram + AdheSE	10.6 (1.5, 0) CDE	12.5(1.8, 0) G	18	<0.05
Gradia Direct P + G-Bond	11.9 (1.5, 0) CDE	18.5 (2.1, 0) F	55	<0.05
BEAUTIFIL II + FL-BOND II	18.4 (4.3, 0) A	27.5 (3.6, 0)	49	<0.05
EPIC-AP + Brush & Bond	9.3 (1.4, 0) DE	16.7 (2.5, 0) FG	80	<0.05
Estelite Sigma + One-Up Bond F Plus	8.7 (1.5, 5) DE	18.2 (3.2, 0) F	109	<0.05
Clearfil AP-X + Clearfil tri-S Bond	17.8 (2.9, 0) AB	20.6 (3.6, 0) F	16	NS

n = 10

^a^: t-test

NS: No significant difference between two conditions (p>0.05). Adh.: Number of adhesive failures. Means with the same letters (A-G) were not significantly different according to Tukey‘s test (p>0.05).

#: Percentage change when compared with the immediate condition

Immediately after setting and after one-day storage, highest bond strengths were exhibited by BEAUTIFIL II. Lowest bond strengths were exhibited by QuiXX, Herculite XRV, Tetric EvoCeram, Gradia Direct P, EPIC-AP, and Estelite Sigma immediately after setting. After one-day storage, lowest bond strengths were exhibited by Herculite XRV, Tetric EvoCeram and EPIC-AP.

At both time points, the number of adhesive failures for each resin composite restorative material was almost zero.

### Shear bond strength to dentin

Table 8 presents the shear bond strengths to dentin. Significant differences (*p*<0.05) in shear bond strength were observed between the immediate time point and after one-day storage for all materials (expect P-60, Tetric EvoCeram, and BEAUTIFIL II), ranging from +18% to +89%.

**Table 8:** Shear bond strengths to dentin (MPa; mean (standard deviation), number of adhesive failures).

Restoration	Immediately	After one-day storage	Change (%) ^#^	p value ^a^
QuiXX + Xeno IV	10.1 (1.4, 0) AB	15.8 (2.4, 0) CD	56	<0.05
P-60 + Adper Prompt L-Pop	9.9 (2.1, 0) AB	11.9 (2.5, 0) D	20	NS
Herculite XRV + AIO Adhesive	10.0 (3.2, 0) A	16.9 (2.8, 0) CE	69	<0.05
Tetric EvoCeram + AdheSE	11.6 (2.6, 0) AB	14.4 (3.3, 0) CD	24	NS
Gradia Direct P + G-Bond	10.6 (1.4, 0) AB	16.3 (2.0, 0) CD	54	<0.05
BEAUTIFIL II + FL-BOND II	16.6 (3.0, 0)	19.6 (2.9, 0) E	18	NS
EPIC-AP + Brush & Bond	10.4 (2.1, 0) AB	16.9 (3.5, 0) C	63	<0.05
Estelite Sigma + One-Up Bond F Plus	8.5 (2.4, 3) B	16.1 (5.1, 0) CD	89	<0.05
Clearfil AP-X + Clearfil tri-S Bond	13.1 (1.2, 0) A	20.4 (3.8, 0) E	56	<0.05

n = 10

^a^: *t*-test

NS: No significant difference between two conditions (p>0.05). Adh.: Number of adhesive failures. Means with the same letters (A-G) were not significantly different according to Tukey‘s test (p>0.05).

^#^: Percentage change when compared with the immediate condition

Immediately after setting and after one-day storage, highest bond strengths were exhibited by BEAUTIFIL II. Lowest bond strengths were exhibited by QuiXX, P-60, Herculite XRV, Tetric EvoCeram, Gradia Direct P, EPIC-AP, and Estelite Sigma immediately after setting. After one-day storage, lowest bond strengths were exhibited by QuiXX, P-60, Tetric EvoCeram, Gradia Direct P, and Estelite Sigma.

At both time points, the number of adhesive failures for each resin composite restorative material was almost zero.

### Flexural properties

Tables 9 and 10 present the flexural strength and modulus data, respectively, obtained at two time points.

**Table 9:** Flexural strengths of restorative materials (MPa; mean (standard deviation)).

Restoration	Immediately	After one-day storage	Change (%) ^#^	p value ^a^
QuiXX + Xeno IV	84.4 (3.3) A	143.8 (12.1) D	70	<0.05
P-60 + Adper Prompt L-Pop	102.0 (5.6)	165.1 (9.8) E	62	<0.05
Herculite XRV + AIO Adhesive	75.5 (9.3) B	135.9 (10.5) DF	80	<0.05
Tetric EvoCeram + AdheSE	84.1 (5.0) A	122.7 (3.5) FG	46	<0.05
Gradia Direct P + G-Bond	52.2 (3.5)	91.5 (7.0) H	75	<0.05
BEAUTIFIL II + FL-BOND II	77.0 (4.9) A	113.9 (11.3) G	4	<0.05
EPIC-AP + Brush & Bond	62.2 (5.0) C	108.6 (10.4) G	75	<0.05
Estelite Sigma + One-Up Bond F Plus	61.9 (5.4) C	93.5 (7.1) H	51	<0.05
Clearfil AP-X + Clearfil tri-S Bond	128.4 (7.6)	167.9 (14.1) E	31	<0.05

n = 10

^a^: *t*-test

NS: No significant difference between two conditions (p>0.05). Means with the same letters (A-G) were not significantly different according to Tukey‘s test (p>0.05).

^#^: Percentage change when compared with the immediate condition

**Table 10:** Flexural moduli of restorative materials (GPa; mean (standard deviation)).

Restoration	Immediately	After one-day storage	Change (%) ^#^	p value ^a^
QuiXX + Xeno IV	9.29 (2.63) A	18.21 (1.71) F	96	<0.05
P-60 + Adper Prompt L-Pop	8.62 (1.24) A	15.76 (1.19)	83	<0.05
Herculite XRV + AIO Adhesive	4.77 (0.13) BC	11.88 (0.70) G	149	<0.05
Tetric EvoCeram + AdheSE	6.04 (0.87) BD	9.21 (0.88)	52	<0.05
Gradia Direct P + G-Bond	2.78 (0.22) E	5.26 (0.31)	89	<0.05
BEAUTIFIL II + FL-BOND II	7.05 (0.86) D	11.78 (0.99) G	67	<0.05
EPIC-AP + Brush & Bond	5.26 (0.50) B	10.77 (0.73) G	105	<0.05
Estelite Sigma + One-Up Bond F Plus	3.59 (0.19) CE	6.88 (0.46)	92	<0.05
Clearfil AP-X + Clearfil tri-S Bond	10.99 (0.98)	17.76 (1.35) F	62	<0.05

n = 10

^a^: *t*-test

NS: No significant difference between two conditions (p>0.05). Means with the same letters (A-G) were not significantly different according to Tukey‘s test (p>0.05).

^#^: Percentage change when compared with the immediate condition

Significant differences (*p*<0.05) in flexural strength were observed between the immediate time point and after one-day storage for all resin composite filling materials, ranging from +31% to +80%. Immediately after setting, Clearfil AP-X showed the highest value while Gradia Direct P showed the lowest. After one-day storage, P-60 and Clearfil AP-X showed the highest values, while Gradia Direct P and Estelite Sigma showed the lowest.

For flexural modulus data, significant differences (*p*<0.05) were observed between the immediate time point and after one-day storage for all restorative materials, ranging from +52% to +149%. Immediately after setting, Clearfil AP-X showed the highest value while Gradia Direct P and Estelite Sigma showed the lowest. After one-day storage, QuiXX and Clearfil AP-X showed the highest values, while Gradia Direct P showed the lowest.Cavity models which mimicked the geometry of typical Class I cavities were used in this study. Although these models provided only morphological simulation, they also provided a consistent, reproducible geometry which is essential for scientific studies. Microscopic analyses of marginal gaps formed at cavosurface margins and interfacial gaps formed internally within the cavities gave a three-dimensional portrayal of gap incidence in a Class I restoration. Statistically lower gap incidence was observed for all composite materials with one-day-delayed polishing when compared with finishing immediately after setting. Therefore, the hypothesis of this study which stated that premature finishing would significantly reduce gap formation when compared with delayed finishing was rejected.

Polymerization shrinkage occurs before or after the gel point; only polymerization shrinkage which occurs after the gel point induces stress. In a cavity, post-gel shrinkage is counteracted by bonding to the cavity walls and plastic flow of the resin. For light-cured materials, they reach their gel points very rapidly [20]. When bonded to dentin in a tooth cavity, polymerization stress of resin-based adhesives reportedly increased only during light curing, reached their maximum shortly after light exposure, but showed a continuous decrease soon after [21, 22]. Since post-gel polymerization shrinkage presents the highest vulnerabilities for gap formation [12, 14, 22], higher bond strength and higher plastic flow would result in lower polymerization stress. This is because these characteristics afford a longer time to resist gap formation and allow smaller gaps to be formed when the latter occurs.

Adhesive agents used in this study exhibited different shear bond strengths when measured immediately after polishing and after one-day storage. Bond strength depends on numerous factors: type of self-adhesive system, pH value, monomer type and concentration, solvent type (such as water or acetone), and adhesive composition (solvent and filler content). In the present study, there was neither moisture nor solvent evaporation control in the bonding procedure [23–29]. Thus, it was likely that some of these factors could have affected shear bond strength results—but it would be difficult to pinpoint the contribution of each factor based on the data obtained.

Self-adhesive systems were used in the present study because of interfacial continuity formed between the cavity walls and adhesive agents, which was accomplished by the simultaneous demineralization and penetration of these agents [28, 30]. This was a definite advantage over the problem of technique sensitivity associated with total-etch bonding systems. With Clearfil AP-X, immediate bond strength to enamel was greater than that to dentin. Subsequently, this adhesive agent showed no significant difference between immediate and one-day-delayed bond strengths to enamel. In the case of Clearfil AP-X, the pH value of its self-etch adhesive agent, Clearfil tri-S Bond, was 2.7 [31]. After etching the enamel substrate, resin tags were created to provide micromechanical retention. We surmised that the immediate bonding to enamel substrate provided by micromechanical retention was greater than that to dentin substrate, which was provided by a created hybrid layer [32].

Most of the fracture modes seen after shear testing were mixed and cohesive failures in resin. Therefore, shear bond strengths to the tooth substrates (enamel and dentin) correlated with the mechanical strength of the filling material or luting agent [14, 17]. The higher bond strength after one-day storage, compared with the immediate condition [33], resulted partly from the stiffer filling materials with higher moduli. In resin composite restorations, early shear bond strength and flexural moduli predominantly affected their early interfacial gap formation and marginal gap-width incidence. Likewise in the Teflon molds, these characteristics also influenced the formation of both interfacial and marginal gaps. After one-day storage, interfacial gap formation and marginal gap-width incidence were significantly reduced compared with the immediate condition [14].

In the present study, commercially available resin composites were used for investigation. Despite significant differences in bonding performance, all composites displayed similar tendencies in their bond strengths to enamel and dentin and in their flexural properties when measured immediately after polishing and after one-day storage. This could be attributed to their similar filler-matrix ratios [34].

For all resin composites examined in this study, the presence and width of interfacial gaps were significantly reduced when restorations were polished after one-day storage. Contributing causes were increased bond strengths to enamel and dentin after one-day storage (Tables 7 and 8) and increased flexural strengths and moduli (Tables 9 and 10). Resin composites were not plagued by the problem of excess water absorption and swelling, which then heightens the risk of gap formation. After one-day water storage, all Class I restorations revealed 14–22 interfacial gaps of 10–30 μm compared with 27–38 interfacial gaps detected immediately after polishing.

Cervical corners of resin composite restorations in this study yielded more gaps than the coronal corners. This was because cervical dentin tended to show lower bond strength than coronal dentin [30].

Greater interfacial integrity exhibited by resin composite restorations in this study could stem from a combination of factors: smaller polymerization shrinkage, lower polymerization shrinkage stress, and good bond strength. In clinical settings, it might be advisable to delay polishing when resin composites are used for Class I restorations since improved mechanical properties were displayed after one-day storage. The clinical implication is that dentists and patients must agree to a next-day return visit for polishing to improve the survival rate of their restorations.

## Conclusions

Commercially available resin composites tested in this study exhibited significantly improved interfacial integrity when Class I restorations were polished after one-day delay as opposed to polishing immediately after setting.
